# Prone Positioning Was Associated With Less Hypoxemic Events and Improved Feeding Tolerance in Preterm Infants

**DOI:** 10.1111/apa.70153

**Published:** 2025-05-26

**Authors:** Bettina Bohnhorst, Eva Lutz, Sabine Pirr, Corinna Peter, Carolin Böhne

**Affiliations:** ^1^ Department of Pediatric Pneumology, Allergology and Neonatology Hannover Medical School Hannover Germany; ^2^ Department of Child and Adolescent Psychiatry and Psychotherapy University Medical Center Hamburg‐Eppendorf Hamburg Germany

**Keywords:** cardiorespiratory events, gastric residuals, preterm

## Abstract

**Aim:**

Cardiorespiratory events such as apnea, bradycardia and hypoxemia are common in preterm infants and may contribute to an impaired neurodevelopmental outcome. We hypothesised a significant reduction in the incidence of hypoxemia, bradycardia and gastric residuals in the prone position.

**Methods:**

In this monocentric, prospective, randomised, two‐arm crossover trial, at a tertiary university hospital, 48 preterm infants with a gestational age of < 32 weeks and a postmenstrual age of < 34 weeks were randomised to either a prone–supine or a supine–prone sequence of cardiorespiratory monitoring over a period of 24 h. The primary outcome parameter was the cumulative frequency of hypoxemias and bradycardias; secondary parameters comprised evaluation of basal parameters, hypoxemias, bradycardias and the amount of gastric residuals.

**Results:**

The cumulative frequency of hypoxemias and bradycardias and the number of severe hypoxemias (peripheral oxygen saturation [SpO_2_] < 80%) were halved in the prone position (*p* = 0.03). Median basal SpO_2_ was significantly higher (*p* = 0.01) and gastric residuals were significantly lower (*p* = 0.0002) in the prone position. The frequency of apneas (> 10 s) was significantly increased in the prone position (*p* = 0.01).

**Conclusion:**

Prone positioning of preterm infants significantly reduces the cumulative frequency of hypoxemias and bradycardias, severe hypoxemias and gastric residuals while increasing basal SpO_2_.

**Abbreviations**
bpm: beats per minute
CPAP: continuous positive airway pressure
CRE: cardiorespiratory events
FiO_2_
: fraction of inspired oxygen
GA: gestational age
NICU: neonatal intensive care unit
pCO_2_
: partial pressure of carbon dioxide
PMA: postmenstrual age
SpO_2_
: peripheral oxygen saturation

**Summary**

- Our trial investigated cardiorespiratory events and gastric residuals in 48 preterm infants < 32 weeks gestational age in relation to body position.
- The frequency of hypoxemias and bradycardias and the number of severe hypoxemias were halved in the prone position; gastric residuals were significantly lower, and median basal SpO_2_ was higher in the prone position.
- Preterm infants up to 34 weeks postmenstrual age may benefit from prone positioning during hospitalisation.

## 1 Introduction

Cardiorespiratory events (CRE) such as apnea, bradycardia and hypoxemia are common in preterm infants. The underlying pathophysiology reflects a multifactorial genesis including immature respiratory control as well as somatic factors [1]. Prolonged and severe hypoxemias have been shown to affect later neurodevelopment and should therefore be prevented [2, 3].

However, the influence of body position on CRE is still inconclusive. Several studies have reported favourable effects of prone positioning on breathing patterns and the incidence of CRE [4, 5]. Some national guidelines recommend prone positioning for preterm infants to reduce CRE [6]. In contrast, a meta‐analysis found no significant effect of different body positions on CRE [7]. Body position also appears to influence feeding tolerance in preterm infants, and several studies have suggested that the prone position is superior to the supine position for reducing gastric residuals [8, 9]. However, the overall evidence for optimal positioning in preterm infants has been of limited quality, and further randomised controlled studies are therefore required [9].

The aim of this study was to conduct a randomised controlled crossover study to assess the frequency and severity of CRE in relation to body position in preterm infants with a gestational age (GA) of less than 32 weeks. We hypothesised that prone positioning reduces hypoxaemias and bradycardias by at least 30%.

## 2 Methods

### 2.1 Study Design and Participants

Between May 2016 and July 2018, we conducted a monocentric, prospective, randomised, two‐arm cross‐over study at Hannover Medical School, Germany, a tertiary neonatal intensive care unit (NICU).

Preterm infants with a GA of < 32 weeks at birth and < 34 weeks postmenstrual age (PMA) at the time of the study with relevant CRE were eligible. Relevant CRE was defined as more than six episodes of hypoxemia < 80% and bradycardia < 80 beats per minute (bpm)/6 h. Infants with congenital malformations, an intestinal pathology, or the need for noninvasive positive pressure ventilation or mechanical ventilation were preexcluded. Secondary exclusion parameters comprised changes of ventilatory support, insufficient monitoring quality during recording, or secondary CRE due to an acute pathology such as infection.

All patients were randomly assigned by a random number generator to either prone (group A) or supine (group B) as the starting position. Each patient spent 12 h in the starting position before being switched. To minimise diurnal and carry‐over effects, a 12‐h washout period in the prone position was performed between the two recordings, based on a previous cross‐over study [10]. Blinding of the patient's position during the intervention was not possible.

Infants were either breathing spontaneously on room air, receiving supplemental oxygen, receiving respiratory support via humidified high‐flow nasal cannula, or receiving continuous positive airway pressure (CPAP). Ventilatory support remained unchanged throughout the study period, with oxygen delivery only being adjusted briefly during prolonged hypoxemia or nursing care to achieve a saturation of 90%–95%.

### 2.2 Cardiorespiratory Event Monitoring and Analysis

VitaGuard 3100 monitoring unit (GETEMED AG, Brandenburg, Germany) was used to record electrocardiogram, thoracic breathing movements and peripheral oxygen saturation (SpO_2_) as previously described [11]. In addition to the total recording time and duration and percentage of artefact‐free recording time, we extracted and further analysed basal heart rate, SpO_2_ and respiratory rate during periods of quiet sleep according to Stebbens et al. [12] The frequency and duration of central apneas lasting ≥ 10 s and the percentage of periodic breathing during quiet sleep were assessed. Frequency, duration and severity of bradycardias < 80 bpm and hypoxemias, the latter defined as moderate SpO_2_ ≤ 85%–80% and severe < 80%, were also recorded. Data were analysed using VitaWin software version 3.3 (GETEMED AG, Brandenburg, Germany). Data extraction and analysis was reviewed by a senior neonatologist blinded to the patient's position during recording.

Nursing staff recorded gastric residuals before each feeding. Depending on the infant's current weight, meals were provided every 2 or 3 h, resulting in six or four feeding time‐points per 12 h, respectively.

### 2.3 Statistical Analysis

The primary outcome parameter was the cumulative frequency of hypoxemias and bradycardias per hour in prone versus supine body position. The secondary outcomes included basal SpO_2_, heart rate and respiratory rate, percentage of periodic breathing, frequency and duration of apneas, moderate and severe hypoxemias, bradycardias < 80 bpm and the proportional amount of gastric residuals from the previous meal. Sample size calculation resulted in 48 patients, based on a previous cross‐over study [10]. A normal distribution of all outcome parameters and a 30% decrease in the primary outcome parameter in the prone position was assumed. We aimed to detect a significant difference with 80% power at the 5% significance level (*p* < 0.05).

Demographic and clinical parameters were recorded. Statistical analysis was performed using GraphPad Prism software (version 10; GraphPad Software, Massachusetts, USA). Following the Shapiro–Wilk normality test for Gaussian distribution, both groups were further analysed, including the paired or unpaired t‐test, Mann–Whitney test or Wilcoxon matched‐pairs rank test, as appropriate. A carry‐over effect (ΔA − ΔB) was tested, with *p* < 0.05 considered significant.

### 2.4 Ethics

Approval of the study protocol was obtained from the local ethics committee (No. 7160). Written informed consent was obtained for each child from their legal guardians. All procedures involving human participants were performed in accordance with the Declaration of Helsinki.

## 3 Results

Forty‐eight patients with a median GA of 26.9 weeks (range 24.4–30.3) and mean birth weight of 884 g (±SD 250 g) were included. At the time of the study, patients had a mean PMA of 31.7 weeks (±SD 1.2) and weight of 1273 g (±SD 276 g) (Table 1). Each subgroup comprised 24 patients with well‐matched demographic parameters, except for median weight at study entry. This was significantly lower in group B (median 1163 g, range 830–1980 g) compared to group A (median 1378 g, range 640–2080 g) (*p* = 0.04) (Table 1). The total number of hypoxemia and bradycardia events was not affected by the starting position (ΔA − ΔB; *p* = 0.43, data not shown). In addition, there was no significant carry‐over effect in any of the secondary outcome parameters analysed (Tables S1 and S2).

**TABLE 1:** Demographic parameters.

Parameters [range]/[SD]	All patients (*n* = 48)	A (*n* = 24)	B (*n* = 24)	*p* (A vs. B)
**Patient data**				
Sex	Male *n* = 28 Female *n* = 20	Male *n* = 12 Female *n* = 12	Male *n* = 16 Female *n* = 8	0.38^1^
Antenatal steroids (yes/no)	Yes: *n* = 46 No: *n* = 2	Yes: *n* = 22 No: *n* = 2	Yes: *n* = 24 No: *n* = 0	0.49^1^
Mode of delivery	S *n* = 6 CS *n* = 42	S *n* = 2 CS *n* = 22	S *n* = 4 CS *n* = 20	0.66^1^
GA at birth (weeks)	26.9^#^ [24.4–30.3]	27.5^+^ [±1.8]	26.9^+^ [±1.8]	0.27^2^
PMA at study (weeks)	31.7^+^ [±1.2]	31.9^+^ [±1.1]	31.5^+^ [±1.4]	0.37^2^
Chronological age at study (days)	33.5^#^ [12.0–58.0]	31.1^+^ [±11.9]	33.2^+^ [±13.8]	0.57^2^
Birth weight (g)	884^+^ [±250]	938^+^ [±268]	830^+^ [±222]	0.13^2^
Weight at study (g)	1273^+^ [±276]	1378^#^ [640–2080]	1163^#^ [830–1980]	*0.04* ^1^
Feeding tube (Ch)	6.0^#^ [4.0–8.0]	6.0^#^ [4.0–8.0]	6.0^#^ [4.0–8.0]	0.65^1^
Position feeding tube (oral/nasal)	Oral: *n* = 42 Nasal: *n* = 6	Oral: *n* = 20 Nasal: *n* = 4	Oral: *n* = 22 Nasal: *n* = 2	0.66^1^
**Respiratory data and medication**				
RDS (grade)	2^#^ [0–4]	2^#^ [1–3]	2^#^ [0–4]	0.09^1^
SIMV duration (day)	0.2^#^ [0–21]	0^#^ [0–21]	1.5^#^ [0–15]	0.23^1^
Resp. support at study	CPAP *n* = 38 HHFNC *n* = 3 Low‐flow *n* = 1 None *n* = 6	CPAP *n* = 17 HHFNC *n* = 2 Low‐flow *n* = 0 None *n* = 5	CPAP *n* = 21 HHFNC *n* = 1 Low‐flow *n* = 1 None *n* = 1	0.14^1^
PEEP at study (cmH_2_O) (CPAP, HHFNC)	5.4^#^ [2.9–7.2]	5.0^#^ [2.9–6.5]	5.6^#^ [4.0–7.2]	0.14^1^
Percentage of oxygen (%)	21.0^#^ [21–34]	21.0^#^ [21–27]	23.0^#^ [21–34]	0.07^1^
Caffeine citrate (yes/no)	Yes: *n* = 46 No: *n* = 2	Yes: *n* = 23 No: *n* = 1	Yes: *n* = 23 No: *n* = 1	> 0.99^1^
Caffeine citrate dosage (mg/kg/day)	5.1^#^ [0–9.7]	5.03^#^ [0–9.3]	5.1^#^ [0–9.7]	0.86^1^
Doxapram at time of study (yes/no)	Yes: *n* = 4 No: *n* = 44	Yes: *n* = 2 No: *n* = 22	Yes: *n* = 2 No: *n* = 22	> 0.99^1^
**Laboratory data**				
pCO_2_ [mmHg]	49.2^+^ [±6.1]	49.7^+^ [±4.2]	48.7^+^ [±7.5]	0.58^2^
Hb [g/dl]	12.5^+^ [±2.5]	12.0^+^ [±2.3]	13.1^+^ [±2.6]	0.12^2^
Hct [%]	37.5^+^ [±7.4]	36.1^+^ [±6.9]	38.9^+^ [±7.8]	0.19^2^
**Complications prior to study**				
Dexamethason (yes/no)	Yes: *n* = 4 No: *n* = 44	Yes: *n* = 1 No: *n* = 23	Yes: *n* = 3 No: *n* = 21	0.61^1^
PDA‐treated by indomethacin (yes/no)	Yes: *n* = 14 No: *n* = 34	Yes: *n* = 6 No: *n* = 18	Yes: *n* = 8 No: *n* = 16	0.75^1^
No IVH IVH grade I/II/III	*n* = 41 *n* = 4/2 / 1	*n* = 20 *n* = 3/1 / 0	*n* = 21 *n* = 1/1/1	> 0.99^1^
Posthemorrhagic hydrocephalus (yes/no)	Yes: *n* = 1 No: *n* = 47	Yes: *n* = 0 No: *n* = 24	Yes: *n* = 1 No: *n* = 23	> 0.99^1^
Sepsis (yes/no)	Yes: *n* = 4 No: *n* = 44	Yes: *n* = 1 No: *n* = 23	Yes: *n* = 3 No: *n* = 21	> 0.99^1^

*Note:* All data are presented as median^#^ (range) or mean^+^ (SD) (according to Shapiro‐Wilk normality test). Other statistical analyses:^1^ Mann‐Whitney test,^2^ unpaired Student's t‐test. Italic indicates the significance level of *p* ≤ 0.05.

Abbreviations: Ch, Charrière; CPAP, continuous positive airway pressure; CS, Caesarean section; Group A, prone‐supine sequence; group B, supine‐prone sequence; GA, gestational age; HHFNC, humidified high‐flow nasal cannula; IVH, intraventricular haemorrhage; PDA, patent ductus arteriosus; PEEP, positive end‐expiratory pressure; PMA, postmenstrual age; RDS, respiratory distress syndrome; S, spontaneous delivery; SIMV, synchronised intermittent mandatory ventilation.

### 3.1 Primary Endpoint

**FIGURE 1:**
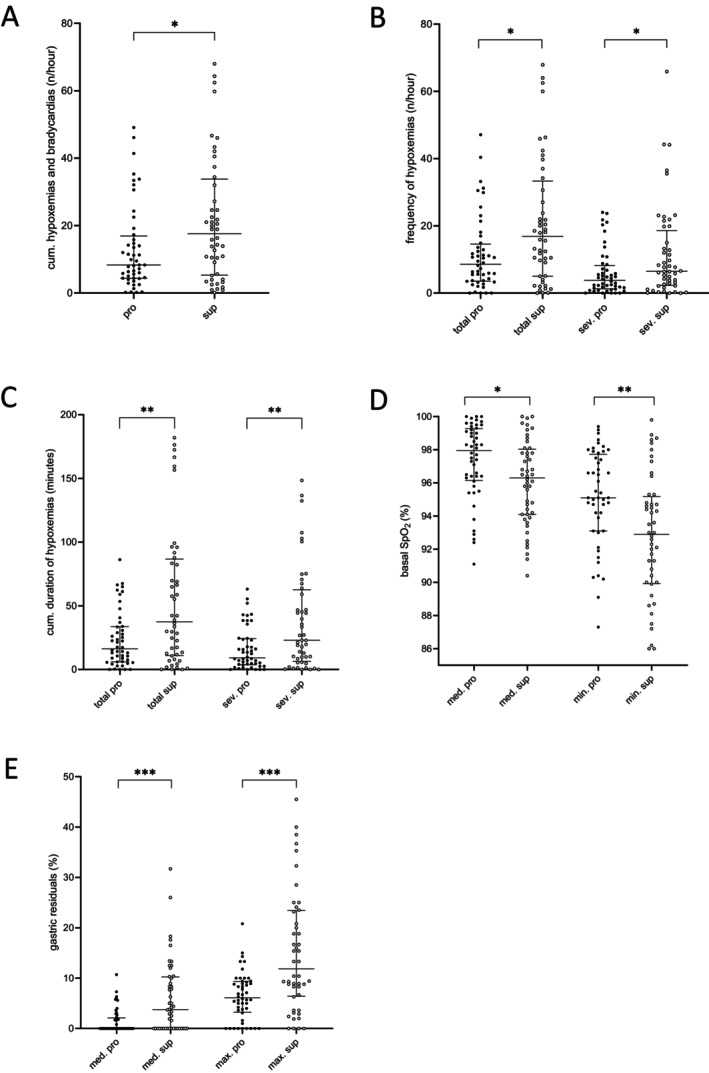
Analyses of cumulative hypoxemias and bradycardias, hypoxemias, basal SpO_2_ and gastric residuals. (A) Total number per hour of cumulative hypoxemias and bradycardias (*n*/h). (B) Frequency of hypoxemias including total (SpO_2_ ≤ 85%) and severe (SpO_2_ < 80%) hypoxemias (*n*/h). (C) Cumulative duration of total (SpO_2_ ≤ 85%) and severe (SpO_2_ < 80%) hypoxemic events (minutes). (D) Median basal SpO_2_ (%) and minimum basal SpO_2_ (%). (E) Median and maximum of gastric residuals of previous meal (%). Dot plots show median and interquartile range. *p ≤ 0.05, **p ≤ 0.01, ***p ≤ 0.001. cum, cumulative; max, maximum; med, median; min, minimum; pro, the prone position; sev, severe (SpO_2_ < 80%); Sup, the supine position.

The cumulative frequency of all hypoxemias and bradycardias per hour was significantly decreased and almost halved in the prone position compared to the supine position (median frequency/h supine *n* = 17.6, prone *n* = 8.3, *p* = 0.03) (Figure 1a and Table 2). This decrease resulted primarily from an almost halved total number of all hypoxemias in the prone position (Figure 1b and Table 2). The positive effect of prone positioning included both moderate, defined as SpO_2_ ≤ 85%–80%, and severe, defined as SpO_2_ < 80% hypoxemias, the latter more clinically relevant (median frequency/h: supine *n* = 6.5, prone *n* = 3.8, *p* = 0.03) (Figure 1b and Table 2). The occurrence of bradycardias did not differ between the two positions (total number/h prone *n* = 0.4, supine *n* = 0.4, *p* = 0.59) (Table S1).

**TABLE 2:** Hypoxemias.

	Total (*n* = 48)		*p*
	Sup	Pro	
**Hypoxemias and bradycardias**			
Total (*n*)	205.0^#^	106.5^#^	*0.02* ^1^
Total/h (*n*)	17.6^#^	8.3^#^	*0.03* ^1^
**Total hypoxemias**			
Total (*n*)	196.5^#^	103.0^#^	*0.02* ^1^
Total/h (*n*)	16.9^#^	8.6^#^	*0.01* ^1^
Cum. total duration (minutes)	37.5^#^	16.3^#^	*0.005* ^‐1^
Median duration/event (seconds)	7.0^#^	6.0^#^	0.28^1^
Median nadir (% SpO_2_)	80.0^#^	80.0^#^	0.65^1^
**Severe hypoxemias**			
Total (*n*)	80.0^#^	45.5^#^	*0.03* ^1^
Total/h (*n*)	6.5^#^	3.8^#^	*0.03* ^1^
Cum. total duration (minutes)	22.9^#^	9.1^#^	*0.007* ^1^
Median duration/event (seconds)	9.0^#^	8.0^#^	0.12^‐^
Median nadir (% SpO_2_)	74.8^+^	75.2^+^	0.28^2^
Min. nadir (% SpO_2_)	50.5^#^	56.9^#^	0.30^1^

*Note:* Median^#^ or mean^+^ (according to Shapiro‐Wilk normality testing) of all hypoxemia (and bradycardia) events depending on group affiliation and followed by^1^ Mann‐Whitney test,^2^ unpaired Student's t‐test. Italic indicates the significance level *p* ≤ 0.05.

Abbreviations: % SpO_2_, % peripheral oxygen saturation; Cum., cumulative; Group A, prone‐supine sequence; group B, supine‐prone sequence.

### 3.2 Secondary Endpoints

Further analyses illustrate the differences in the frequency and severity of hypoxemias between the two positions (Figure 1 and Table 2). The total time infants spent in hypoxemia was significantly decreased and more than halved in the prone position (cumulative duration total supine 37.5 min, prone 16.3 min, *p* = 0.005) (Figure 1c and Table 2). This finding applied in particular to the subgroup of severe hypoxemias (cumulative duration severe supine 22.9 min, prone 9.1 min, *p* = 0.007) (Figure 1c and Table 2). In addition, we saw a trend toward a worsening of the severity of hypoxemias in the supine position by as much as 6% (minimum nadir severe supine 50.5%, prone 56.9%, *p* = 0.30) (Table 2). However, the median duration per event was not significantly different between the two positions (supine 7.0 s, prone 6.0 s, *p* = 0.28) (Table 2).

Basal parameters for all 48 patients during quiet sleep such as median heart rate, respiratory rate, the occurrence of periodic breathing or supplemental oxygen delivery did not differ significantly between prone and supine positions (Tables S1 and S2). In contrast, both median and minimum basal SpO_2_ were significantly increased in prone positioning (median SpO_2_ supine 96.3%, prone 97.9%, *p* = 0.01; min. SpO_2_ supine 92.9%, prone 95.1%, *p* = 0.002) (Figure 1d and Table S2).

The proportion of gastric residuals from the previous meal was significantly lower in the prone than in the supine position, though not reaching the estimated 20% threshold (median gastric residuals% supine 3.75%, prone 0.0%, *p* = 0.0002*;* max. gastric residuals % supine 11.9%, prone 6.1%, *p* = 0.0001) (Figure 1e and Table S2).

Interestingly, the frequency of apneas was significantly increased in the prone position compared to the supine position (median number/h prone *n* = 1.2, supine *n* = 0.4, *p = 0.01*). However, the median duration/event did not differ between both positions (supine 10.2 s, prone 11.0 s, *p* = 0.08) (Table S1).

## 4 Discussion

Our study involving 48 well‐characterised preterm infants is one of the largest studies to date assessing the influence of body position on CRE in very low birth weight infants with a median GA of 27 weeks and a mean PMA of 32 weeks. The cumulative frequency of hypoxemias and bradycardias, the primary outcome, was significantly decreased and almost halved in the prone position. Detailed analyses attributed this increase primarily to the higher frequency of hypoxemias, as the occurrence of bradycardias did not differ between both positions. However, bradycardias are the least frequent event in extremely premature infants and do not appear to affect long‐term neurocognitive outcomes [13]. Furthermore, severe hypoxemic events with SpO_2_ < 80% not only occurred half as often in the prone position but also their total duration was more than halved. This emphasises the clinical relevance of our results. Surprisingly, prone positioning significantly increased the frequency of apneas. In addition to all cardiorespiratory parameters, a significant decrease in gastric residuals was observed in the prone position. The latter highlights another clinically important consideration.

Several studies have investigated the impact of body position on CRE and the occurrence of gastric residuals in preterm infants, with varying results. In general, prone positioning may optimise lung function and breathing patterns by increasing PaO_2_ [14], tidal volume, thoraco‐abdominal synchrony [15], and total sleep duration [16]. However, a meta‐analysis of spontaneously breathing preterm infants concluded that these studies were insufficient to determine the effect of body position on CRE in these patients. The authors attributed this to small study numbers and sample sizes [7]. Our data therefore add substantially to the ongoing debate.

We found a 1.6% higher basal mean SpO_2_ during quiet sleep in prone compared to supine positioning, which is consistent with a meta‐analysis that demonstrated a trend toward a 1.9% increase in mean SpO_2_ in CPAP‐ventilated neonates [17]. In addition, supine positioning in extremely preterm infants resulted in an increased need for supplemental oxygen to maintain target oxygen saturation, independent of PMA [18]. Although we could not confirm a reduction of supplemental oxygen in our study, prone positioning contributed to a reduction in hypoxemic events. In comparative studies of different SpO_2_ target ranges, very immature preterm infants randomised to a target saturation of 91%–95% had less intermittent hypoxemias compared to those randomised to a target range of 85%–89%. However, the duration and severity of such events were comparable between both groups [19]. Our data support these findings, since the duration per event and also the median nadir were comparable in both positions.

Our results are further consistent with a longitudinal study by Shepherd et al. who demonstrated a reduced frequency and shorter duration of desaturations and bradycardias in extremely preterm infants during the prone position, regardless of PMA. They concluded that CRE are predominantly defined by the GA at birth, rather than the current PMA [18]. Accordingly, current SIDS prevention guidelines from the American Academy of Paediatrics recommend placing preterm infants in the supine position from 32 weeks PMA. This allows infants to get used to this position before discharge and thereby reduces the risk for unsafe sleeping practices [20]. Prolonged prone positioning may counteract these efforts. In addition, one has to consider that prolonged prone positioning may result in an increased risk for an unfavourable musculoskeletal outcome with hypertonia in the lower limbs [21]. However, our data suggest that individualised timing of supine positioning of preterm infants to a later PMA and respiratory stability may be more beneficial. This potentially minimises the need for more invasive interventions or medications during hospitalisation.

Surprisingly, our data revealed that prone positioning significantly increased the number of apneas. This is in line with a study by Bhat et al., which described an increased frequency and duration of central apneas ≥ 5 s in the prone position [16]. Dimitriou et al. hypothesised that a more relaxed breathing in the prone position leads to a higher number of apneas [22]. In contrast, a review by Picheansathian et al. confirmed a decreased incidence of apnea in the prone position, together with improved arterial oxygen saturation and lung and chest wall synchrony [5]. Interestingly, Bhat et al. [16] also reported a significant increase in obstructive and mixed apneas in the *supine* position.

Since we have only analysed apneas with a duration of ≥ 10 s, this may have resulted in the underreporting of shorter apneas in the supine position. Nevertheless, these shorter apneas are clinically irrelevant and merely an expression of premature breathing patterns, provided that they are not accompanied by severe hypoxemias. Second, by recording thoracic breathing movements, we were only able to detect central apneas. However, in preterm infants, it is uncommon to distinguish between central and obstructive apneas. Central apneas frequently lead to upper airway collapse, reflecting the key mechanism for the resulting hypoxemias and bradycardias [1]. Due to the decreased distance between the threshold of regular breathing and apneas in preterm infants, a small decrease in partial pressure of carbon dioxide (pCO_2_) may increase the risk of inducing apneas [23]. Despite all speculation, the increase in apneas in the prone position observed is not of clinical relevance because they were neither of longer duration nor accompanied by hypoxemia. Neurodevelopment is influenced by the frequency, severity and duration of hypoxemia rather than the number or duration of apneas [13].

Body position not only influenced cardiorespiratory stability but also affected feeding tolerance in preterm infants, as shown in a meta‐analysis by Halemani et al. [24] The authors found significantly less gastric residuals due to accelerated gastric emptying in infants placed in the prone or the right lateral positions compared to the supine and the left lateral positions. This reduction in gastric residuals may also influence the incidence of CRE by reducing gastric distension and consequently enhancing lung volume. The latter was supported by Richmond et al. who showed that *propped and prone—*positioning significantly reduced CRE by 50%. In addition, *propped and prone—*positioning improved the percentage of time with a SpO_2_ ≥ 88% compared to supine positioning regardless of feeding mode [25]. In our study, we also observed a significant reduction of proportional gastric residuals in the prone position. Although the absolute amount of gastric residuals was very small and well below the clinically relevant amount of 20% of the feeding volume. However, in accordance with current literature [26], we should emphasise that routine gastric residuals testing is controversial and is generally no longer recommended in our department.

## 5 Limitations

Our study has several limitations. First, data were collected from a single NICU, limiting the total sample size, although we still included one of the largest groups of patients. Second, patients’ weight at study entry was slightly but significantly different in both groups. However, the cross‐over design of our study ensured that each patient served as his/her own control and thereby minimised the potential influence of this one parameter. Lastly, missing data on pCO_2_ levels affect the interpretation of apnea results, although this was not one of our outcome parameters.

## 6 Conclusion

Our data show that very preterm infants may benefit from prone positioning at least until 34 weeks PMA, due to a reduced frequency of hypoxemias, and thus a reduced need for supplemental medication during hospital stay. It is absolutely reasonable to apply current guidelines and accustom premature infants to the supine position in time for discharge to monitor individual cardiorespiratory stability under real life conditions. However, it may be useful to individualise the timing of this step according to the infant's GA and PMA and their respiratory stability.

## Author Contributions

**Bettina Bohnhorst:** conceptualisation, methodology, data acquisition, formal analysis, validation, writing – original draft and review and editing; **Eva Lutz:** data acquisition, formal analysis, investigation and writing – review and editing; **Sabine Pirr:** formal analysis, validation and writing – review and editing; **Corinna Peter:** validation, writing – review and editing; **Carolin Böhne:** formal analysis, validation, writing – original draft and writing – review and editing.

## Conflicts of Interest

The authors declare no conflicts of interest.

## Supporting information


**Table S1.** Data of bradycardias, apneas and periodic breathing. Median^#^ or mean^+^ (according to Shapiro–Wilk normality testing) of all bradycardias, apneas and periodic breathing depending on group affiliation and followed by ^1^paired Student t‐test, ^2^Mann–Whitney test, ^3^unpaired Student t‐test, ^4^Wilcoxon matched‐pairs rank test; significance level *p* = 0.05.

**Table S2.** Basal parameters. Median^#^ or mean^+^ (according to Shapiro–Wilk normality testing) of all basal parameters depending on group affiliation and followed by ^1^paired Student t‐test, ^2^Mann–Whitney test, ^3^unpaired Student t‐test, ^4^Wilcoxon matched‐pairs rank test; significance level *p* = 0.05.

## Data Availability

All data generated or analysed during this study are included in this article and its Tables S1 and S2 files. Further enquiries can be directed to the corresponding author.
