# Supplementary material for: Prone Positioning Was Associated With Less Hypoxemic Events and Improved Feeding Tolerance in Preterm Infants
Source: Acta Paediatr. 2025 May 26;114(10):2643–50. doi: 10.1111/apa.70153 (PMC12420861; doi:10.1111/apa.70153)
Supplement: Supplementary file 1 — Table S1. Data of bradycardias, apneas and periodic breathing. Median# or mean+ (according to Shapiro–Wilk normality testing) of all bradycardias, apneas and periodic breathing depending on group affiliation and followed by 1paired Student t‐test, 2Mann–Whitney test, 3unpaired Student t‐test, 4Wilcoxon matched‐pairs rank test; significance level p = 0.05. [file APA-114-2643-s002.docx]

**Table S1: Data of bradycardias, apneas and periodic breathing.**

|  | **A**  **(n=24)** | | | | **p-value** | | **B**  **(n=24)** | | | **p-value** | **ΔA vs. ΔB**  **p-value** | **total**  **(n=48)** | | **p-value** | | |
| --- | --- | --- | --- | --- | --- | --- | --- | --- | --- | --- | --- | --- | --- | --- | --- | --- |
|  | **sup** | | | **pro** | |  | **sup** | | **pro** |  |  | **sup** | **pro** |  |  |  |
| **bradycardias** |  | | | | |  |  | | |  |  |  | |  | | |
| total (n) | 6.0^#^ | | | 6.0^#^ | | 0.67^4^ | 7.0^#^ | 3.5^#^ | | 0.29^4^ | 0.13^2^ | 6.0^#^ | 5.0^#^ | 0.51^2^ | | |
| total/hour (n) | 0.2^#^ | | | 0.5^#^ | | 0.37^4^ | 0.6^#^ | 0.2^#^ | | ***0.03^4^*** | 0.34^2^ | 0.4^#^ | 0.4^#^ | 0.59^2^ | | |
| cum. total duration (minutes) | 0.6^#^ | | | 1.0^#^ | | 0.18^4^ | 1.0^#^ | 0.5^#^ | | 0.14^4^ | ***0.04^2^*** | 0.8^#^ | 0.9^#^ | 0.91^2^ | | |
| med. duration/event (seconds) | 6.5^#^ | | | 9.0^#^ | | ***0.006^4^*** | 9.0^#^ | 7.5^#^ | | 0.85^4^ | ***0.02^3^*** | 7.0^#^ | 8.0^#^ | 0.16^2^ | | |
| med. nadir (bpm) | 66.7^#^ | | | 66.0^#^ | | 0.66^4^ | 64.5^#^ | 67.0^#^ | | 0.26^4^ | 0.21^3^ | 65.0^#^ | 66.0^#^ | 0.95^2^ | | |
| min. nadir (bpm) | 49.7^+^ | | | 50.2^+^ | | 0.86^1^ | 48.3^+^ | 52.7^+^ | | 0.23^1^ | 0.38^3^ | 52.5^#^ | 52.0^#^ | 0.76^2^ | | |
|  |  |  | | |  | |  | |  |  |  |  |  |  | | |
| **apneas** |  | | | | |  |  | | |  |  |  | |  | | |
| total (n) | 5.5^#^ | | | 6.0^#^ | | 0.12^4^ | 4.0^#^ | 17.0^#^ | | ***<0.0001^4^*** | 0.13^2^ | 4.5^#^ | 14.5^#^ | ***0.01^2^*** | | |
| total/hour (n) | 0.4^#^ | | | 0.6^#^ | | 0.12^4^ | 0.3^#^ | 1.5^#^ | | ***0.0007^4^*** | 0.17^2^ | 0.4^#^ | 1.2^#^ | ***0.01^2^*** | | |
| cum. total duration (minutes) | 0.9^#^ | | | 1.6^#^ | | 0.15^4^ | 0.7^#^ | 3.7^#^ | | ***<0.0001^4^*** | 0.12^2^ | 0.8^#^ | 3.1^#^ | ***0.01^2^*** | | |
| med. duration/event (seconds) | 11.0^#^ | | | 11.2^#^ | | 0.33^4^ | 11.0^#^ | 11.0^#^ | | 0.21^4^ | 0.92^2^ | 10.2^#^ | 11.0^#^ | 0.08^2^ | | |
|  |  |  | | |  | |  | |  |  |  |  |  |  | | |
| **periodic breathing** |  | | | |  | |  | | |  |  |  | |  | |  |
| total (n) | 12.5^#^ | | 7.5^#^ | | 0.2^4^ | | 6.0^#^ | 5.5^#^ | | 0.94^4^ | 0.31^2^ | 7.5^#^ | 6.0^#^ | 0.69^2^ | | |
| total/hour (n) | 1.0^#^ | | 0.6^#^ | | 0.29^4^ | | 0.5^#^ | 0.5^#^ | | 0.9^4^ | 0.39^2^ | 0.6^#^ | 0.5^#^ | 0.78^2^ | | |
| cum. total duration (minutes) | 8.5^#^ | | 5.2^#^ | | 0.32^4^ | | 4.8^#^ | 4.2^#^ | | 0.83^4^ | 0.37^2^ | 5.4^#^ | 4.8^#^ | 0.76^2^ | | |
| med. duration/event (seconds) | 41.4^+^ | | 40.6^+^ | | 0.85^1^ | | 40.5^#^ | 38.7^#^ | | 0.36^4^ | 0.21^2^ | 40.9^#^ | 41.2^#^ | 0.99^3^ | | |

*Group A = prone-supine sequence, group B = supine-prone sequence. ΔA, ΔB: differences for matched pairs of group A or B. Cum.: cumulative; med.: median; min.: minimum; bpm: beats per minute.*
