# Supplementary material for: Prone Positioning Was Associated With Less Hypoxemic Events and Improved Feeding Tolerance in Preterm Infants
Source: Acta Paediatr. 2025 May 26;114(10):2643–50. doi: 10.1111/apa.70153 (PMC12420861; doi:10.1111/apa.70153)
Supplement: Supplementary file 2 — Table S2. Basal parameters. Median# or mean+ (according to Shapiro–Wilk normality testing) of all basal parameters depending on group affiliation and followed by 1paired Student t‐test, 2Mann–Whitney test, 3unpaired Student t‐test, 4Wilcoxon matched‐pairs rank test; significance level p = 0.05. [file APA-114-2643-s001.docx]

**Table S2: Basal parameters.**

|  | **A**  **(n=24)** | | **p-value** | **B**  **(n=24)** | | **p-value** | **ΔA vs. ΔB**  **p-value** | **total**  **(n=48)** | | | **p-value** | | |
| --- | --- | --- | --- | --- | --- | --- | --- | --- | --- | --- | --- | --- | --- |
|  | **sup** | **pro** |  | **sup** | **pro** |  |  | **sup** | | **pro** |  | | |
| **heart rate (bpm)**  **median** | 155.0^+^ | 157.0^+^ | 0.08^1^ | 158.8^+^ | 160.1^+^ | 0.34^1^ | 0.91^2^ | 156.9^+^ | | 158.6^+^ | | 0.35^3^ | |
| **min.** | 149.3^+^ | 150.8^+^ | 0.24^1^ | 152.4^+^ | 154.9^+^ | 0.05^4^ | 0.61^3^ | 150.8^+^ | | 152.8^+^ | | 0.28^3^ | |
| **max.** | 161.7^+^ | 165.6^+^ | ***0.03***^1^ | 168.0^#^ | 167.0^#^ | 0.46^4^ | 0.38^3^ | 163.8^+^ | | 166.6^+^ | | 0.14^3^ | |
| **respiratory rate (brpm)**  **median** | 67.7^+^ | 67.9^+^ | 0.93^1^ | 68.5^+^ | 69.4^+^ | 0.68^1^ | 0.86^2^ | 68.1^+^ | | 68.7^+^ | | 0.82^3^ | |
| **min.** | 51.5^#^ | 54.0^#^ | 0.83^4^ | 58.8^+^ | 58.3^+^ | 0.80^1^ | 0.80^3^ | 56.0^#^ | | 55.0^#^ | | 0.80^2^ | |
| **max.** | 78.0^#^ | 76.0^#^ | 0.42^4^ | 81.0^+^ | 80.8^+^ | 0.93^1^ | 0.45^3^ | 79.8^+^ | | 80.9^+^ | | 0.65^3^ | |
| **SpO_2_ (%)**  **median** | 96.0^#^ | 98.2^#^ | ***0.03^4^*** | 96.5^#^ | 97.6^#^ | ***0.02^4^*** | 0.87^3^ | 96.3^#^ | | 97.9^#^ | | ***0.01****^2^* | |
| **min.** | 93.9^#^ | 95.3^#^ | ***0.03^4^*** | 92.3^+^ | 95.1^+^ | ***0.001^1^*** | 0.50^3^ | 92.9^#^ | | 95.1^#^ | | ***0.002****^2^* | |
| **max.** | 98.8^#^ | 99.5^#^ | 0.36^4^ | 98.5^#^ | 98.9^#^ | 0.08^4^ | 0.63^3^ | 98.6^#^ | | 99.3^#^ | | 0.07^2^ | |
| **FiO_2_**  **median** | 0.21^#^ | 0.21^#^ | >0.99^4^ | 0.23^#^ | 0.23^#^ | 0.50^4^ | >0.99^2^ | 0.21^#^ | | 0.21^#^ | | 0.82^2^ | |
| **min.** | 0.21^#^ | 0.21^#^ | >0.99^4^ | 0.23^#^ | 0.23^#^ | 0.50^4^ | 0.45^2^ | 0.21^#^ | | 0.21^#^ | | 0.99^2^ | |
| **max.** | 0.21^#^ | 0.21^#^ | 0.25^4^ | 0.23^#^ | 0.24^#^ | >0.99^4^ | 0.41^2^ | 0.22^#^ | | 0.21^#^ | | 0.83^2^ | |
| **gastric residuals (%)**  **median** | 4.1^#^ | 0.0^#^ | ***0.0007^4^*** | 2.9^#^ | 0.0^#^ | ***0.0006^4^*** | 0.82^2^ | 3.75^#^ | | 0.0^#^ | | ***0.0002****^2^* | |
| **max.** | 0.0^#^ | 0.0^#^ | 0.11^4^ | 0.0^#^ | 0.0^#^ | 0.23^4^ | 0.62^2^ | 0.0^#^ | | 0.0^#^ | | ***0.02^2^*** | |
| **max.** | 9.1^#^ | 5.5^#^ | ***0.002^4^*** | 14.3^#^ | 7.0^#^ | ***0.0002^4^*** | 0.55^2^ | 11.9^#^ | | 6.1^#^ | | ***0.0001****^2^* | |
| **gastric residuals (ml)**  **median** | 0.95^#^ | 0.0^#^ | ***0.0015****^4^* | 0.5^#^ | 0.0^#^ | ***0.0015***^4^ | 0.82^2^ | 0.85^#^ | | 0.0^#^ | | ***0.0002****^2^* | |
| **min.** | 0.0^#^ | 0.0^#^ | 0.11*^4^* | 0.0^#^ | 0.0^#^ | 0.39^4^ | 0.54^2^ | 0.0^#^ | | 0.0^#^ | | ***0.03****^2^* | |
| **max.** | 2.5^#^ | 1.2^#^ | ***0.002****^4^* | 2.0^#^ | 1.1^#^ | ***0.0003***^4^ | 0.93^2^ | 2.5^#^ | 1.1^#^ | | | | ***<0.0001****^2^* |

*Group A = prone-supine sequence, group B = supine-prone sequence. ΔA, ΔB: differences for matched pairs of group A or B. SpO_2_: peripheral oxygen saturation; FiO_2_: fraction of inspired oxygen; bpm: beats per minute; brpm: breaths per minute; min.: minimum; max: maximum.*
